# Is the Wedged Insole an Effective Treatment Option When Compared with a Flat (Placebo) Insole: A Systematic Review and Meta-Analysis

**DOI:** 10.1155/2018/8654107

**Published:** 2018-12-04

**Authors:** Bingbing Zhang, Xing Yu, Long Liang, Liguo Zhu, Xiaopeng Dong, Yang Xiong, Quan Pan, Yongsheng Sun

**Affiliations:** ^1^Dongzhimen Hospital, Beijing University of Chinese Medicine, Beijing 100700, China; ^2^Wangjing Hospital of China Academy of Chinese Medical Sciences, Beijing 100102, China; ^3^First Affiliated Hospital of Anhui University of Chinese Medicine, Anhui 230031, China; ^4^Dezhou People's Hospital, Dezhou, Shandong 253000, China

## Abstract

**Background:**

Using the lateral wedge insole is a conservative management strategy for knee osteoarthritis. The theoretical basis for this intervention is to correct femorotibial angle, thereby reducing pain and optimising function.

**Objective:**

This systematic review evaluates the evidence on the effectiveness of wedge insole compared with flat insole for the treatment of knee osteoarthritis.

**Methods:**

A systematic review was performed, searching published (MEDLINE, EMBASE, CNKI, Cochrane Library, and Web of Science) and unpublished literature from their inception to April 2018. Randomized controlled trials (RCTs) that compared the use of wedge insole with a flat insole were included. Risk of bias and clinical relevance were assessed, and outcomes were analysed through meta-analysis.

**Result:**

From a total of 413 citations, 8 studies adhered to the a priori eligibility criteria. The WOMAC pain was shown to be statistically nonsignificant change with the use of wedge insole (SMD=0.07), and low heterogeneity (I^2^=22%) and a 95% CI that crossed zero (95% CI: −0.09 to 0.24). The 5 independent trials were not significant in improving pain score (SMD = −0.02, 95% CI: −0.19 to 0.16). This review also revealed no significance in improving Lequesne index (SMD = −0.27, 95% CI: −0.72 to 0.19). The meta-analysis from the 2 independent trials was significant in improving femorotibial angle (SMD = −0.41, 95% CI: −0.73 to -0.09). In conclusion, this meta-analysis suggested that lateral wedge insoles can improve femorotibial angle but are of no benefit with pain and functions in knee osteoarthritis.

## 1. Introduction

Knee osteoarthritis (KOA), as the most common musculoskeletal disorders, is a degenerative articular joint disease which leads to erosion and degradation of the articular cartilage, formation of extended bone, and narrowing of the joint space [1, 2]. In general, KOA is associated with symptoms such as knee stiffness, pain around knee, and restricted movement range of knee joint [3]. It is a leading cause of disability among older individuals aged above 40 years. Besides affecting patients' activity and quality of life, OA will further cause depression and anxiety, as well as being a great economic burden [4]. In China, the prevalence of radiographic KOA was 42.8% in women and 21.5% in men (prevalence ratio 1.45), and the prevalence of KOA in Chinese men was similar to that in their white US counterparts (prevalence ratio 0.90) [5].

Many factors are related to the occurrence of knee osteoarthritis, such as aging, obesity, increased knee joint movement, low income, and relevant multiple metabolic disorders, which are important associated factors for KOA [6]. Although there are many methods for treating knee osteoarthritis, their effects are limited [7–9]. Usually, the conservative treatment of patients with KOA is aimed at altering the biomechanics of the knee to reduce the knee joint load, relieve symptoms, and slow progression of KOA in cases of knee malalignment [10]. Osteoarthritis typically affects joints in a nonuniform manner [11]. The predominance of medial compartment osteoarthritis likely results from the high medial compartment forces during weight-bearing activities such as walking [11]. The previous researches show that the normal forces acting on the leg produce a varus torque during walking. This varus torque is directly associated with the compressive force across the medial aspect of the knee, which is nearly 2.5 times the force through the lateral aspect of the knee [12, 13]. Knee varus torque is believed to be responsible for the progression of knee osteoarthritis. Many clinical data has already proved that knee pain and disease progression can be relieved by the tibial osteotomy which increases lateral loading and effectively reduces the knee varus torque [14–16]. Whether the conservative means can achieve the same effect, the wedge insole, an inexpensive intervention for potentially altering knee joint biomechanics, might be of interest in the treatment of KOA [17].

Wedge insole is a wedge placed under the sole of the shoe, regardless of how it is placed, so that it is thicker at the lateral part than other area and an angle is formed. Thus, lateral wedge insole can change the toe out angle. Hurwitz's research [18] shows that the toe out angle was predictive of the peak adduction moment (R = -0.45, p < 0.001). By gait analysis, Hurwitz et al. also find that the peak external knee adduction moments in subjects with KOA were correlated with the mechanical axis of the leg, usually measured by the femorotibial angle (FTA), (R=0.74, p<0.001) [18]. A study including several similar literatures has also proved this relationship by meta-analysis [19]. So it may transfer load from medial to lateral knee joints during weight-bearing. Many studies [20–22] have documented wedge insole can effectively relieve pain, improve knee function, and improve the femorotibial angle. Nevertheless, some literatures [23] suggest that it cannot significantly improve the pain and function of KOA. In addition, the guidelines on KOA developed by different groups have different opinions on wedge insole for the treatment of KOA, such as the American College of Rheumatology and the Osteoarthritis Research Society [24].

Although the previous meta-analyses have reported on the same topic, different conclusions were drawn due to the difference in the control group [17, 19, 25–27]. The objective of this article was to assess the efficacy of lateral wedge insole (LWI) treatments compared with flat (neutral) insole (FI) for patients with KOA by assessing pain, function, and FTA as a marker for mediolateral load shift, reported in randomized controlled trials (RCTs) that we can retrieve in line with the established criteria. By assessing RCTs comparing two different insoles, LWI and FI, we want to explore whether the insole only acts as a placebo in the treatment of KOA.

## 2. Methods

The review protocol was registered with the International Prospective Register of Systematic Reviews (PROSPERO registration no. CRD42018094547), available online: http://www.crd.york.ac.uk/PROSPERO/display_record.php?ID=CRD42018094547). All pooled analyses are based on previously published studies, and thus no ethical approval and patient consent are required.

### 2.1. Literature Research

As with the original review, we used the search strategies recommended by the Cochrane Back Review Group for the identification of RCTs [32]. Trails were retrieved from PubMed, Embase, Web of Science, Cochrane library, and CNKI. We searched for all relevant articles published from inception of each database until April, 2018. There were no limits on study dates or any language, publication type, and status restrictions. The key terms used in these searches were “Knee Osteoarthritis”, “Osteoarthritis of the knee”, “Knee Osteoarthritides”, “Osteoarthritides, Knee”, “Osteoarthritis of Knee”, “Knee, Osteoarthritis of”, “Knees, Osteoarthritis of”, “Osteoarthritis Of Knees”, “orthoses, Foot”, “Foot Orthosis”, “Orthosis, Foot”, “Foot Orthotic Devices”, “Device, Foot Orthotic”, “Devices, Foot Orthotic”, “Foot Orthotic Device”, “Orthotic Device, Foot, Orthotic Devices, Foot, Foot Arch Supports, Arch Support, Foot”, “Foot Arch, Insole”, “Arch Supports, Foot”, “Foot Arch Support, Insole”, “Orthotic Shoe Inserts”, “Insert, Orthotic Shoe”, “Inserts, Orthotic Shoe”, “Orthotic Shoe Insert”, “Shoe Insert, Orthotic”, “Shoe Inserts, Orthotic”, “Orthotic Insoles”, “Insole, Orthotic”, “Insoles, Orthotic”, and “Orthotic Insole”. In addition, the reference lists of previously published systematic reviews on the subject of wedge insole for the treatment of KOA were manually examined for pertinent studies.

### 2.2. Inclusion Criteria

According to the requirements of the Cochrane manual [32], trails were screened by 2 independent investigators to evaluate eligibility, and any discrepancies were resolved by discussion or further evaluated by the third one. First, the titles and abstracts of searched studies were screened. Then, full papers were reviewed to examine whether each study met the following criteria: (1) they were randomized controlled trial; (2) type of participants must be patients suffering from KOA; (3) experimental studies should be using wedge insole (control group includes flat insole, neutral insole); (4) outcomes should include one of WOMAC, pain, femorotibial angle (FTA), and Lequesne index. When multiple time points were reported either in one particular report of a study or over the course of several articles from the same study, the longest follow-up period on treatment was considered in our article. If overlapping subject populations were enrolled in different reports, the one of higher quality or with a larger sample size was selected for inclusion.

### 2.3. Exclusion Criteria

The studies were excluded due to the following reasons: (1) studies does not conform to the above criteria; (2) both the treatment group and the control group included wedge insole therapy for KOA; (3) studies were in the form of letters, abstracts, reviews, or comments; (4) studies were impossible to extract relevant data; and (5) the KOA patients were treated with surgery.

### 2.4. Data Extraction

Four authors extracted data. Authors extracted the following information using a predesigned collection form: the first author's name, year of publication, study type, country, number of patients under wedge insole treatment and control group, BMI index, age of patients, and the time point. Information on outcomes of interest including WOMAC, pain, Lequesne index, and FTA of the patients was also collected and extracted. We contacted authors of original study for additional data when necessary.

### 2.5. Quality Assessment

We assessed the risk of bias of RCTs in this review using the Cochrane risk of bias tool [32]. For each included study, each type of bias was rated as high, low, or unclear and entered into the risk of bias table. Four review authors, two with methodological expertise and two with content expertise, independently assessed the risk of bias of the included studies. The review authors resolved any disagreements by discussion, including input from a third independent review author if required.

### 2.6. Grading the Quality of Evidence

The Grading of Recommendations Assessment, Development, and Evaluation (GRADE) method was used to assess the quality of the evidence for each outcome of meta-analysis. Levels of quality of evidence recommended by the GRADE Working Group were defined as high(++++), moderate(+++), low(++), and very low(+). The judgments were based on risk of bias, inconsistency, indirectness, imprecision, and publication bias. We operated on this web page:https://gradepro.org/.

### 2.7. Data Synthesis and Statistical Analysis

Data regarding outcomes in the eligible trials were combined in the meta-analysis using the RevMan 5.3 software (Cochrane Collaboration, Oxford, United Kingdom). Since the results of interest are continuous variables, the authors calculated weighted mean differences (WMD) to assess the difference between the groups. Also, standardized mean difference (SMD) is chosen if clinical outcome is the same, but different measured methods are used in different trials. Its corresponding 95% confidence interval (CI) for each parameter was computed in wedge insole-treated versus control group. We quantified statistical heterogeneity using the I-squared statistic (I^2^); statistical heterogeneity between the trials was significant when I^2^ > 50% [33]. A fixed-effects model was used to generate the SMD with its corresponding 95% CI if there was no significant heterogeneity of the data (I^2^ < 50%). Otherwise, random-effects model was used if significant heterogeneity existed (I^2^ >50%). Sensitivity and subgroup analysis would then be carried out to assess the robustness of results of meta-analysis for primary outcome.

## 3. Result

### 3.1. Literature Search and Study Sample Characteristics

**Figure 1:**
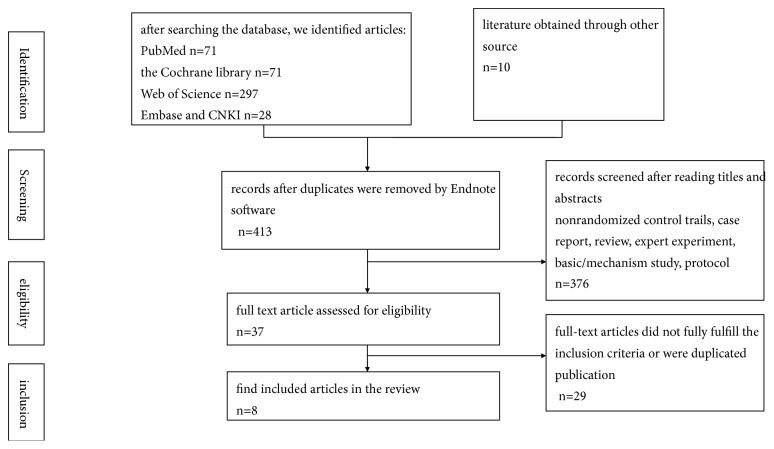
Flow diagram of included studies in the meta-analysis.

We used the outlined literature search strategy and removed duplicates (see Figure 1). We found 477 articles. Of these, 440 were excluded. More than half of these excluded articles (213 articles) were reports of studies of other unrelated orthoses or surgical trials and 227 were not trials (i.e., narrative reviews and systematic, clinical guidance documents, press releases, letters, and commentaries). This left 37 articles assessed after reading whole article for eligibility. Of these, 8 met the inclusion criteria [20–23, 28–31] (see Table 1).

The examined intervention in this review was a lateral wedge insole. Control/comparison conditions are flat (neutral) insole. Only one study [31] examined lateral wedges and arch support. A total of 4 different outcome variables were identified in this review. The Western Ontario and McMaster Universities (WOMAC) Osteoarthritis Indexes that were reported in 4 studies [21, 23, 29, 30] included the WOMAC pain (4 studies) [21, 23, 29, 30], WOMAC stiffness (3/4 studies) [21, 23, 30], and WOMAC function (3/4 studies) [21, 23, 30]; pain was reported in 5 studies [20, 23, 28, 29, 31]; Lequesne index was reported in 3 studies [20, 22, 29]; and FTA was report in 2 studies [20, 22]. According to the different intervention time of the wedge insole, Toda et al. [22] divided the treatment components into three subgroups compared with the flat insole. This study considered that all the 3 subgroups met the inclusion criteria. Therefore, all three subgroups were included in this meta-analysis.

### 3.2. Risk of Bias

**Figure 2:**
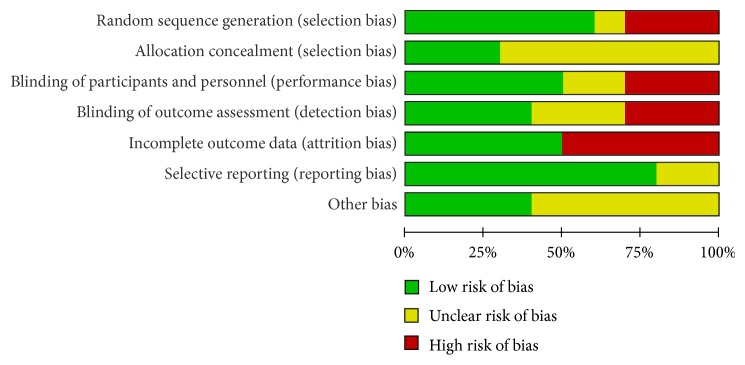
Risk of bias graph: review authors' judgments about each risk of bias item presented as percentages across all included studies.

**Figure 3:**
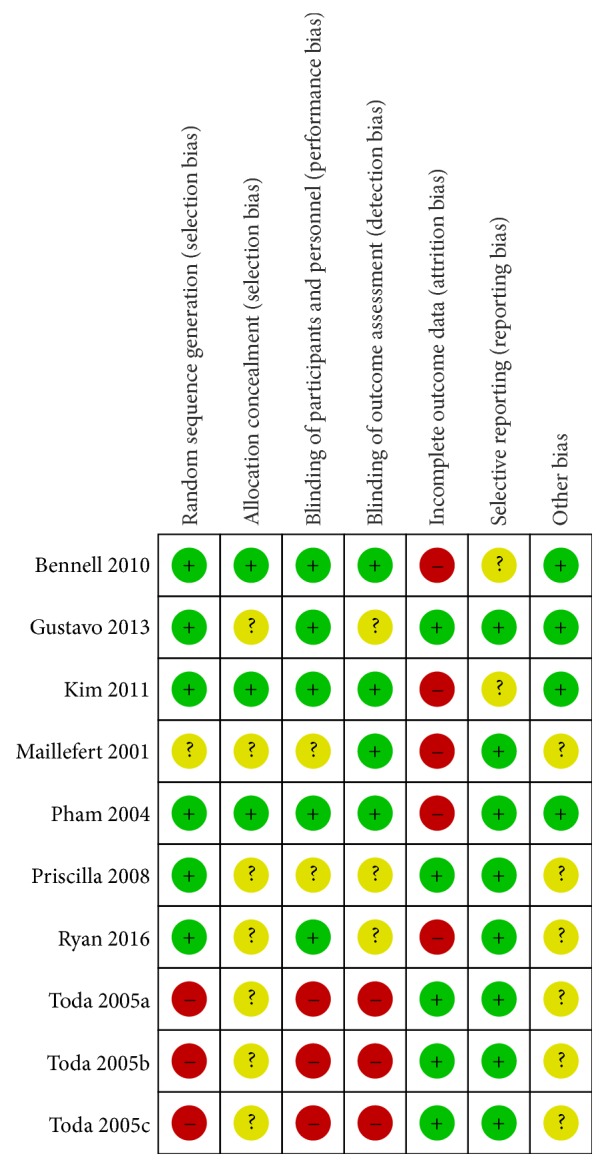
Risk of bias summary: review authors' judgments about each risk of bias item for each included study.

Figures 2 and 3 showed the summary of methodological quality, respectively. In the included studies, six studies clearly described the method used to generate the randomization sequence. One study reported allocation by date of birth [22]. These divided into groups by random order were considered as low risk. Three studies showed allocation concealment [23, 28, 29]. The other studies did not report it clearly. Blinding was applied to five studies [23, 28–31]. The blinding of outcome assessment was reported in four trials [21, 23, 29, 30]. All the trials reported the follow-up data on the outcome. Due to the length of follow-up, shedding patients were reported in 5 studies [21, 23, 29–31]. All of the researches do the Intentionality (ITT) analysis. Baseline imbalance was not found in the demographic characteristics or the outcomes between the study groups.

### 3.3. WOMAC Osteoarthritis Index

**Figure 4:**
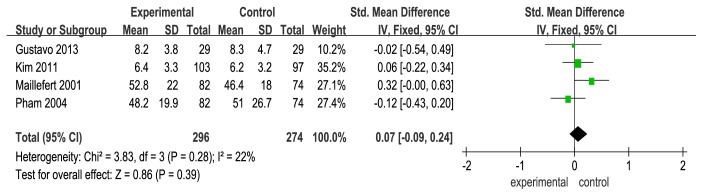
Forest plot of the comparison of wedge insole versus flat insole for WOMAC pain index.

**Figure 5:**
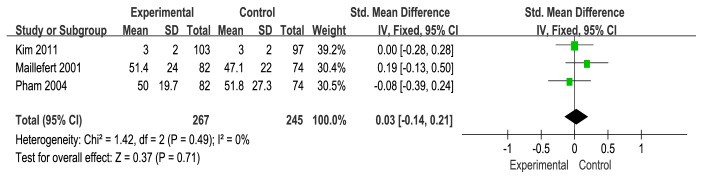
Forest plot of the comparison of wedge insole versus flat insole for WOMAC stiffness index.

**Figure 6:**
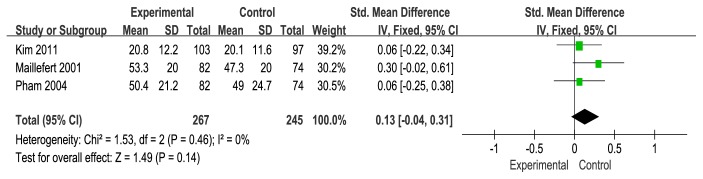
Forest plot of the comparison of wedge insole versus flat insole for WOMAC function index.

Firstly, we examined WOMAC Osteoarthritis Index [34] in this review. WOMAC Osteoarthritis Indexes that were reported in 4 studies [21, 23, 29, 30] included the WOMAC pain (4 studies) [21, 23, 29, 30], WOMAC stiffness (3/4 studies) [21, 23, 30], and WOMAC function (3/5 studies) [21, 23, 30]. A fixed-effects model was used for statistical analysis according to the low heterogeneity (I^2^<50%). The WOMAC pain was shown to be statistically nonsignificant change with the use of wedge insole (SMD=0.07), and low heterogeneity (I^2^=22%) and a 95% CI that crossed zero (95% CI: −0.09 to 0.24) (see Figure 4). The stiffness in the 3 studies was shown to be statistically nonsignificant change with the use of wedge insole (SMD=0.03, 95% CI: −0.14 to 0.21,I^2^=0%, p=0.71) (see Figure 5). The function across 3 studies (SMD=0.13, 95% CI: −0.04 to 0.31, I^2^=0%) provided evidence of statistically nonsignificant change effect between wedge insole and flat insole on the KOA (see Figure 6). Overall, WOMAC indexes provide no significant change in the pain, stiffness, and function.

### 3.4. Pain Scale

**Figure 7:**
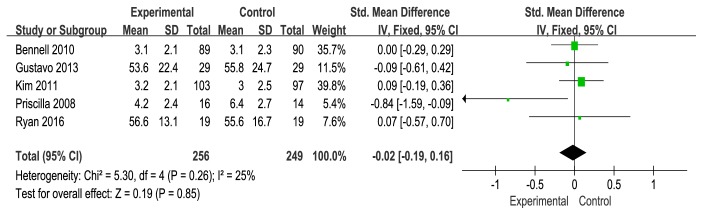
Forest plot of the comparison of wedge insole versus flat insole for pain score.

Five studies reported pain scale [20, 23, 28, 29, 31]. A fixed-effects model was used for statistical analysis according to the low heterogeneity (I^2^ = 25%). The meta-analysis from the 5 independent trials was not significant in improving pain score (SMD = −0.02, 95% CI: −0.19 to 0.16). There was no statistically significant difference in pain score (see Figure 7).

### 3.5. Lequesne Index

**Figure 8:**
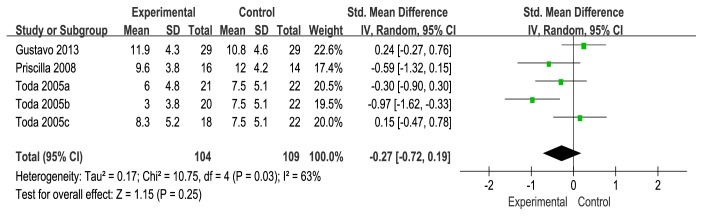
Forest plot of the comparison of wedge insole versus flat insole for Lequesne index.

Three studies [20, 22, 29] compared wedge insole to flat insole in Lequesne index [35]. The meta-analysis from the 3 independent trials was not significant in improving Lequesne index (SMD=−0.27, 95%CI: −0.72 to 0.19). And heterogeneity was high across trial findings (I^2^=63%); a random-effects model was used for statistical analysis. There was no statistically significant difference between the wedge and flat insole groups with respect to Lequesne index (see Figure 8).

### 3.6. Femorotibial Angle (FTA)

**Figure 9:**
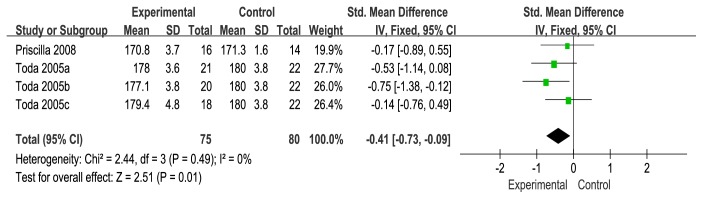
Forest plot of the comparison of wedge insole versus flat insole for femorotibial angle.

FTA was reported in two studies [20, 22]. According to the no heterogeneity (I^2^ = 0%), a fixed-effects model was used for statistical analysis. The meta-analysis from the two independent trials was significant in improving FTA (SMD = −0.41, 95% CI: −0.73 to -0.09). There was statistically significant difference between the use of a wedge and flat insole in FTA [36] (see Figure 9).

### 3.7. GRADE

The GRADE level of evidence is moderate for pain score but low for WOMAC, Lequesne index, and FTA.  Table 3 shows the GRADE evidence profiles. The main reasons for a deceasing level were inconsistency and risk of bias.

## 4. Discussion

This study only included randomized control trails as the analysis target, which compared wedge insole with flat insole. All included subjects are characterized by low heterogeneity and low bias. Eight trails involving 898 participants were included. Patients who received wedge insoles treatment show no significant or clinicallyimportant improvement compared with those who received flat insole treatment in knee pain. But lateral wedge insole can increase FTA, thereby improving knee varus deformity.

The literature included in this study is generally less heterogeneous. But in the group of Lequesne evaluation, the heterogeneity was high. This group included only three studies [20, 22, 29]. And Toda et al. [22] designed a three-arm experiment to compare different wedge angles with flat insole. So, we speculated that the heterogeneity may be related to the differences in wedge angle. Because only 3 studies were included, subgroup analysis was not carried out in this study. The report results were stable when we changed the statistical model to a random effect model. In the sensitivity analysis, the pooled outcomes were stable when we excluded articles one by one and compared the differences of the combining effect before and after exclusion.

WOMAC and Lequesne as evaluation indexes of KOA are recommended by most knee osteoarthritis guidelines [37, 38]. Therefore, this study used WOMAC and Lequesne Index as indicators of the outcome of knee osteoarthritis. Both indicators in this study indicate that there is no significant improvement in lateral wedge insole compared to flat insole. We found that in some articles [39–41], researchers who used no intervention as a control condition reported larger treatment effects than those who opted to use a flat (neutral) insole. Parkes et al. [25] also noticed this result in their study. They speculated that the possible explanation for this finding is that the change in pain caused by the wedge insole may be due to a placebo effect. But there is no clear conclusion. Therefore, studies [29, 31] continued to explore the role of wedge insole in the treatment of KOA after Parkes et al.'s study. This study only included randomized control trails comparing lateral wedge insole with flat insole. The two insoles are very similar and can reduce the selective bias and play a role in controlling the placebo effect [42, 43]. So lateral wedges are no more efficacious than neutral inserts for improvement of pain and function because their effect on the KOA is similar to placebo. And researches [44] show that placebo is effective in the treatment of OA, especially for pain, stiffness, and self-reported function.

This research also shows that wedge insoles produce small increases in FTA to reduce knee varus angle—an important biomechanical outcome associated with knee osteoarthritis progression. Although the meta-analysis from the two independent trials revealed statistical significance in improving FTA, this difference is very small (SMD = −0.41, 95% CI: −0.73 to -0.09). Several previous researches show that lateral wedges cause only 5% to 6% reductions in the knee external adduction moment [45, 46]. According to Hurwitz's research, the FTA were strongly correlated with the knee external adduction moments, (R=0.74, p<0.001) [18], so based on these changes in FTA, changes in the medio-tibial load distribution could be expected. Nevertheless, this may be insufficient to reduce pain.

Although some studies have shown a positive relation between severity of pain and knee load [47, 48], others have found no such relation [18, 49] or even an inverse one [50, 51]. The multiple mechanisms contributing to the experience of pain with knee osteoarthritis possibly explain our results. For example, pain is influenced by a myriad of psychosocial factors that can vary between people as well as within people over time [52]. Hence it is not surprising that favorable biomechanical changes do not guarantee pain reduction.

Although some studies have shown a positive correlation between the severity of pain and knee load [47, 48], other studies have not found this relation [18, 49], or even the opposite one [50, 51]. Multiple risk factors leading to pain in osteoarthritis of the knee may explain our findings. For example, pain is influenced by age, previous knee injuries, and psychosocial elements of work [53]. Therefore, it is not surprising that favorable biomechanical changes do not guarantee pain relief.

This review has limitations. First, we placed randomized control trail on selected studies that reduced the total number of studies, which may have prevented data pooling for some variables. Second, we just limited the analysis to studies that examined lateral wedge insole compared with neutral insole, but we placed no restrictions on disease severity. Finally, additional well-structured higher level studies are required for a more powerful conclusive meta-analysis in the future.

Several previous meta-analyses have reported on the same topic, as presented in Table 2. Differences between the present meta-analysis and the previous ones are as follows. Firstly, our study only compared LWI with FI, ruling out the placebo effect, and the results were more convincing. Our systematic review retrieved Chinese databases and included more literatures to compare LWI with FI. As the latest and most comprehensively updated meta-analysis, the present study further reinforces the earlier results of previous meta-analyses. Secondly, the protocol of this study was registered on PROSPERO. A registered protocol may increase the transparency and quality of meta-analysis. Third, our study included FTA as an outcome indicator. We found a reduction in varus angle when wedged insoles are used, but this may be insufficient to reduce pain. Moreover, the GRADE approach was performed to give the level of evidence. Thus, the conclusions of this study can be clinically used and easily transferred to guidelines.

## 5. Conclusion

In conclusion, the low evidence suggests that lateral wedge insoles can reduce the knee varus angle, but lateral wedges are no more efficacious than neutral inserts for improvement of pain and function in subjects with knee osteoarthritis.

## Figures and Tables

**Table 1:** Basic characteristics of the included trials.

Study ID	Kellgren and Lawrence (K&L)	Age(yrs) Exp/cont	Sample size Exp/cont	BMI	Intervention		Time point	Main outcomes
					Experiment group	Control group		
Kim 2011 [23] Australia	II:95 III:105	63.3±8.1/65±7.9	103/97	28.1±4.2/30.4±5.6	lateral wedge insoles	flat control insoles	12 months	Pain(VAS), WOMAC

Bennell 2010 [28] Australia	NA	NA	89/90	NA	lateral wedge insoles	flat control insoles	12 months	Average pain(VAS)

Gustavo 2015 [29] Brazil	I:4 II:19 III:13 IV:22	65.2±9.6/63.3±7.5	29/29	30.8±6.1/30.3±5.1	lateral wedge insoles with subtalar strapping	a neutral insole with subtalar strapping	24 weeks	WOMAC, VAS, Lequesne index

Maillefert 2001 [21] France	II:73 III:64 IV:19	64±10.8/65.6±9.9	82/74	29±5.6/28.5±5.3	laterally-wedged insoles	neutrally-wedged insoles	6 months	WOMAC

Priscilla 2008 [20] Brazil	II:16 III:8 IV:6	61.6±11.4/61.9±11.3	16/14	28.9±3.5/30.6±3.1	insoles with medial elevation	similar insole without elevation	2 weeks	WOMAC, VAS, Lequesne index, Femorotibial angle

Pham 2004 [30] France	NA	64±10.8/65.6±9.9	82/74	29±5.6/28.5±5.3	Laterally-wedged insoles	Neutrally-wedged insoles	2 years	WOMAC

Ryan 2016 [31] Canada	I:9 II:6 III:5 IV:18	59.9±7.4/59.6±7.7	19/19	32.5±8/29.2±6.7	wedged insoles	Flat insoles	3 months	KOOS pain score

Toda 2005^a^ [22] Japan	NA	63.6±9.9/62±9.8	21/22	24.5±4 /25.5±4.3	lateral wedge with subtalar strapping	subtalar strapping band without lateral wedge	2 weeks	Lequesne index, Femorotibial angle

Toda 2005^b^ Japan	NA	64.1±12.3/62±9.8	20/22	23.8±3.2/25.5±4.3	lateral wedge with subtalar strapping	subtalar strapping band without lateral wedge	2 weeks	Lequesne index, Femorotibial angle

Toda 2005^c^ Japan	NA	64.7±9.7/62±9.8	18/22	24.2±2.3/25.5±4.3	lateral wedge with subtalar strapping	subtalar strapping band without lateral wedge	2 weeks	Lequesne index, Femorotibial angle,

WOMAC =Western Ontario and McMaster Osteoarthritis Index; VAS = visual analogue scale.

**Table 2:** Comparison with other previous meta-analyses.

Author	Parkes /2013 [25]	Penny/2013 [27]	Duivenvoorden/2015 [26]	Xing/2017 [19]	Shaw/2017 [17]	The present meta-analysis
Type of Study	RCTs	RCTs	RCTs	RCTs, quasi-RCTs, cohort studies	Prospective studies	RCTs

Comparison	LWI vs FI LWI vs No treatment	LWI vs FI LWI vs No treatment LWI vs Brace	Valgus brace vs neutral brace LWI vs FI LWI vs no treatment	Shoe-only LWI vs FI (with arch support)	LWI with arch support vs Medial arch supports	LWI vs FI

Number of RCTs(LWIvsFI)	7	7	3	2	3	8

Search strategy until(year)	2013	2012	2014	2016	2016	2018

Protocol registered	NA	NA	Applied	Applied	NA	Applied

Number of search databases	9	5	3	5	4	5

GRADE	NA	NA	Applied	NA	NA	Applied

Outcome Index	WOMAC Pain	WOMAC pain WOMAC physical function Lequesne Index Analgesic consumption Adherence to treatment	Pain scores WOMAC stiffness scores WOMAC function scores Quality of life scores	First peak EKAM Second peak EKAM	Ankle/subtalar eversion angle External ankle eversion moments	WOMAC pain WOMAC stiffness WOMAC function Pain scale Lequesne index Femorotibial angle (FTA)

LWI=lateral wedge insole, FI=flat(neutral) insole.

**Table 3:** Summary of the evidence for each outcome.

**Lateral wedge insole compared to flat(neutral)insole for knee osteoarthritis**					
**Outcomes**	**Anticipated absolute effects** ^**∗**^ ** (95**%** CI)**		№** of participants (studies)**	**Certainty of the evidence ** **(GRADE)**	**Comments**
	**Risk with flat(neutral)insole**	**Risk with lateral wedge insole**			
WOMAC pain follow up: mean 6 months	The mean WOMAC pain was **29.38** SMD	The mean WOMAC pain in the intervention group was 0.07 SMD higher (0.09 lower to 0.24 higher)	570 (4 RCTs)	*⨁⨁*◯◯ LOW ^a^	WOMAC pain (SMD=0.07, 95% CI: −0.09, 0.24), Not statistically significant

WOMAC stiffness index follow up: mean 6 months	The mean WOMAC stiffness index was **31.06** SMD	The mean WOMAC stiffness index in the intervention group was 0.03 SMD higher (0.14 lower to 0.21 higher)	512 (3 RCTs)	*⨁⨁*◯◯ LOW ^a,b^	WOMAC stiffness (SMD=0.03, 95% CI: −0.14, 0.21). Not statistically significant

WOMAC Function follow up: mean 6 months	The mean WOMAC Function was **37.04** SMD	The mean WOMAC Function in the intervention group was 0.13 SMD higher (0.04 lower to 0.31 higher)	512 (3 RCTs)	*⨁⨁*◯◯ LOW ^a,b^	WOMAC function (SMD=0.13, 95% CI: −0.04, 0.31). Not statistically significant

Pain score assessed with: VAS follow up: mean 6 months	The mean pain score was **13.39** SMD	The mean pain score in the intervention group was 0.02 SMD lower (0.19 lower to 0.16 higher)	505 (5 RCTs)	*⨁⨁⨁*◯ MODERATE ^a^	Pain scale (SMD = −0.02, 95% CI: −0.19, 0.16) Not statistically significant

Lequesne index follow up: mean 6 months	The mean Lequesne index was **9.94** SD	The mean Lequesne index in the intervention group was 0.27 SD lower (0.72 lower to 0.19 higher)	213 (3 RCTs)	*⨁⨁*◯◯ LOW ^a,b^	Lequesne index (SMD=−0.27, 95% CI:−0.72, 0.19) Not statistically significant

Femorotibial angle follow up: mean 2 weeks	The mean femorotibial angle was **176.62** SD	The mean femorotibial angle in the intervention group was 0.41 SD lower (0.73 lower to 0.09 lower)	155 (2 RCTs)	*⨁⨁*◯◯ LOW ^a,c^	Femorotibial angle (FTA) (SMD = −0.41, 95% CI:−0.73, -0.09). Statistically significant

*∗* **The risk in the intervention group** (and its 95% confidence interval) is based on the assumed risk in the comparison group and the **relative effect** of the intervention (and its 95% CI).
